# Physical Qualities Pertaining to Shorter and Longer Change-of-Direction Speed Test Performance in Men and Women

**DOI:** 10.3390/sports7020045

**Published:** 2019-02-16

**Authors:** Robert G. Lockie, Brett K. Post, J. Jay Dawes

**Affiliations:** 1 Department of Kinesiology, California State University, Fullerton, CA 92831, USA; brpost@fullerton.edu; 2 College of Nursing and Health Sciences, University of Colorado-Colorado Springs, Colorado Springs, CO 90022, USA; jdawes@uccs.edu

**Keywords:** 505, agility, COD deficit, collegiate club-sport, Illinois agility test, isometric midthigh pull, linear speed, multidirectional jumping, power-to-body mass ratio, tactical athlete

## Abstract

This study investigated relationships between shorter (505, change-of-direction (COD) deficit as a derived physical quality) and longer (Illinois agility test; IAT) COD tests with linear speed, lower-body power (multidirectional jumping), and strength in recreationally-trained individuals. Twenty-one males and 22 females (similar to collegiate club-sport and tactical athletes) were assessed in: 505 and COD deficit from each leg; IAT; 20 m sprint; vertical jump (VJ height, peak anaerobic power measured in watts (PAPw), power-to-body mass ratio); standing broad jump; lateral jump (LJ) from each leg; and absolute and relative isometric midthigh pull (IMTP) strength. Partial correlations calculated sex-determined relationships between the COD and performance tests, with regression equations calculated (*p* < 0.05). The 505 and IAT correlated with all tests except PAPw and absolute IMTP (*r* = ±0.43–0.71). COD deficit correlated with the LJ (*r* = −0.34–0.60). Left- and right-leg 505 was predicted by sex, 20 m sprint, and left-leg LJ (70–77% explained variance). Right-leg COD deficit was predicted by sex and left-leg LJ (27% explained variance). IAT was predicted by sex, 20 m sprint, right-leg LJ, and relative IMTP (84% explained variance). For individuals with limited training time, improving linear speed, and relative lower-body power and strength, could enhance shorter and longer COD performance.

## 1. Introduction

Change-of-direction (COD) speed and agility are essential components in a wide range of sports and activities. Agility has been defined as the initiation of a direction change in response to a stimulus [1]. COD ability or speed is the physical component of agility, which encompasses linear sprinting, technique, lower-body strength and power, and the ability to effectively decelerate and accelerate [1,2,3]. When measuring COD speed, it is important to note that this quality can be task-specific [4], and coaches must interpret this quality within the context of the assessment that was utilized. For example, Lockie [5] detailed that COD speed tests can be grouped under shorter or longer distance, and these can have different applications for coaches and athletes.

An example of a shorter distance COD speed test is the 505 [5]. The 505 involves a 10 m sprint past a timing gate, a further 5 m sprint to a turning line where the individual completes a 180° turn/direction change, before sprinting back through the gate. There has been some analysis of the important underlying physiological characteristics associated with the 505. Delaney et al. [6] found that linear speed measured by a 40 m sprint and relative strength measured by a three-repetition maximum back squat predicted dominant leg 505 time in elite male rugby league players. These researchers also discovered that vertical jump (VJ) and lateral jump (LJ) performance was predictive of 505 performance on the non-dominant leg. Linear speed over 10 m correlated with 505 performance among Division I and II collegiate female soccer players (correlation coefficient *r* = 0.39–0.55) [7]. Lower-body power, measured by a VJ, has also been related to the 505 in collegiate female soccer players (*r* = −0.63 to −0.66) [7]. The standing broad jump (SBJ) related to left-leg 505 in collegiate women rugby union players (*r* = −0.71) [8]. Likewise, relative isometric midthigh pull (IMTP) strength related to 505 performance in female basketball players (*r* = −0.79) [9]. As can be seen, different qualities have been identified as important depending on the sample used. However, there has been limited analysis of combined male and female samples representative of collegiate club-sport athlete [8,10,11,12] and tactical (first responders and military) recruit [13,14,15,16,17,18] populations. Although COD testing can be state- and agency-specific in tactical populations, it does feature in the hiring process of some agencies [19,20,21,22,23]. This is notable, as these individuals may not have the training time afforded to higher-level athletes [24,25,26,27,28]. Accordingly, it is important to understand what characteristics could crossover to shorter distance COD performance in collegiate club-sport and tactical athletes. It is incumbent on these individuals to efficiently improve COD speed pertaining to sport or occupational tasks (e.g., foot pursuits for police) [19,20,21,22,23].

The 505 can be used to produce another measure of COD ability, the COD deficit [29]. This measure calculates the impact that implementing a 180° turn has on the ability to cover a 10 m distance. Previous research has documented non-significant relationships between the COD deficit and linear speed in athletic populations [10,29,30]. In contrast, Lockie et al. [7] found that for both Division I and II collegiate female soccer players, there was a negative relationship between COD deficit and a 10 m sprint (*r* = −0.77 to −0.87). In Division I collegiate male soccer players, the VJ, triple hop, or SBJ did not significantly relate to the COD deficit for either leg [31]. For adolescent males and females, a range of lower-body strength and power assessments correlated with the left- and right-leg COD deficit [32]. Similar to the 505, different physical qualities may be important as it pertains to the COD deficit depending on the sample. However, the relationships between linear speed and lower-body power and strength to the COD deficit requires more analysis.

Lockie [5] described longer distance COD tests as having a duration greater than 6 s. These tests tend to have a greater volume of linear sprinting, interspersed with direction changes. It is important to note that longer distance COD speed tests may be limited by metabolic capacities, as opposed to just COD ability [33]. Nonetheless, these tests are commonly used, with one example being the Illinois agility test (IAT) [5]. The IAT has been used to assess athletic [33,34,35,36,37,38,39,40], law enforcement/public safety [41,42,43], and military [44,45,46] populations. Due to the greater volume of sprinting, a longer distance COD speed test such as the IAT may demonstrate stronger relationships with linear sprinting ability and lower-body multidirectional (vertical, horizontal, and lateral) power measured by jumping [12,47,48]. In occupational testing, longer distance COD speed tests are often used to measure multiple qualities (i.e., linear and COD speed in combination with lower-body power) [19,20,21,22,23]. Analyzing the physical characteristics of men and women to document those most important for longer distance COD speed is useful; this could help improve training efficiency for collegiate club-sport and tactical athletes.

Therefore, this study investigated the relationships between linear speed, lower-body power, and absolute and relative strength on shorter (505, COD deficit) and longer (IAT) COD speed tests in college-aged men and women. The 505 was used as it can isolate COD speed for each leg [6,7,8,9,49,50,51,52,53,54], and can also be used to produce the COD deficit as an alternate measure of COD ability [7,10,29,30,31,32,54]. The IAT was adopted as a longer COD speed test due to its widespread use in athletic [33,34,35,36,37,38,39,40] and tactical [41,42,43,44,45,46] populations. It was hypothesized that when controlling for sex, all variables would correlate with COD performance measured by the 505, COD deficit, and IAT. However, the relationships for linear speed, and lower-body power and strength, would be stronger in the longer COD speed test of the IAT.

## 2. Materials and Methods

### 2.1. Subjects

A convenience sample comprised of 43 subjects (age: 23.14 ± 2.37 years; body mass: 74.32 ± 12.65 kg; height: 1.70 ± 0.08 m), including 21 males (age: 23.38 ± 2.44 years; body mass: 81.38 ± 10.09 kg; height: 1.76 ± 0.06 m) and 22 females (age: 22.91 ± 2.33 years; body mass: 67.59 ± 11.22 kg; height: 1.65 ± 0.07 m). Subjects were recruited from the student population at the university. This sample has utility, as it is similar to collegiate club-sport athletes (mean age: ~21–22 years; male mean body mass and height: ~76–83 kg and ~1.72–1.82 m, respectively; female mean body mass and height: ~62–68 kg and ~1.60–1.63 m, respectively) [8,10,11,12], law enforcement recruits (mean age: ~23–25 years; male mean body mass and height: ~82–85 kg and ~1.80 m, respectively; female mean body mass and height: ~58–68 kg and ~1.67 m, respectively) [13,14], and military recruits (mean data combined for males and females; age: ~20–23 years; body mass: ~59–81 kg; height: ~1.63–1.73 m) [15,16,17,18]. As noted, this pertains to the age ranges across these studies, and the body masses and heights for both males and females. Data were combined for males and females, although as will be detailed, data analysis was controlled for sex. The subjects were required to be: over 18 years of age; currently training in either aerobic or resistance training (≥3 h per week); and free from any musculoskeletal disorders that would influence their ability to participate in the study. Accordingly, the subjects recruited were recreationally active, and familiar to the requirements of the assessments in this study. G*Power software (Version 3.1, Universität Kiel, Kiel, Germany) was used to confirm that for a correlation, point biserial model, a sample size of 43 ensured the data could be interpreted with a moderate (0.40) effect level [55], when the power level was 0.80 and significance was set at 0.05 [56]. Permission to conduct this research was approved by the institutional ethics committee. Subjects received an explanation of the study, which included the risks and benefits of participation, and written informed consent was obtained prior to testing.

### 2.2. Procedures

Prior to data collection, the subject’s age, height, and body mass were recorded. Height was measured barefoot using a stadiometer (Detecto ProDoc, Webb, MO, USA), while body mass was recorded using an electronic digital scale (Ohaus Corporation, Parsippany, NJ, USA). All data were collected across two sessions separated by 24–48 h depending on subject availability. During the first session, each subject completed two trials of the 20 m sprint, SBJ, and LJ (two trials per leg). During the second session, each subject completed two trials of the IAT, 505 (two trials per leg), VJ, and IMTP. The tests were structured in this way to limit fatigue influencing any aspect of test performance, laboratory and equipment availability, and to follow established guidelines for testing order [57]. All sprint and COD tests, in addition to the SBJ and LJ, were completed on a basketball court. Due to equipment availability, the VJ was performed in a laboratory with a concrete floor. The IMTP was performed in the laboratory on top of a force plate. Two minutes recovery was provided between trials and between tests throughout both sessions. The best trial (i.e., fastest sprint or COD test, best jump, highest IMTP force output) was used. Time was recorded to 0.01 s for all speed tests. 

Prior to each testing session, all subjects completed a standardized warm-up, which consisted of approximately 10 min of dynamic stretching of the lower limbs, and linear and lateral runs over 10–20 m that progressively increased in intensity. Subjects were tested at the same time of day for both testing sessions, did not eat for 2–3 h prior to their sessions, and refrained from intensive exercise in the day prior to testing. Subjects were permitted to consume water as required throughout the two sessions.

### 2.3. 20 m Sprint

Linear speed was measured by a 20 m sprint with timing gates (Brower Timing Systems, Draper, UT, USA). Gates were positioned at 0, 5, 10, and 20 m to measure the 0–5 m, 0–10 m, and 0–20 m intervals. These intervals have been used in previous research to indicate linear speed [34,52,53]. The timing gate system was single-beam, and systems such as this have been shown to record reliable data [58,59,60]. Gate height was set at 0.93 m, which follows recommendations from the literature that single-beam gates should be positioned at the approximate hip height for the test subjects [59,61]. Gates were positioned 2.84 m apart, and subjects began the sprint from a standing start 50 cm behind the start line to trigger the first gate. Subjects were instructed to initiate the sprint when ready and cover the set distance as fast as possible. Approximately 15–20 m of extra distance was available after the 20 m mark to ensure subjects sprinted through the last gate, and to provide a safe deceleration zone following the sprint.

### 2.4. Standing Broad Jump (SBJ)

The SBJ was used to indirectly measure lower-body power in the horizontal plane This test was performed according to established methods that have been described in great detail elsewhere [8,31,52,53].

### 2.5. Lateral Jump (LJ)

The LJ was used to indirectly measure lower-body power in the lateral plane and was performed according to established procedures. The methods for the LJ have been described by Lockie et al. [52] and Lockie et al. [53].

### 2.6. Illinois Agility Test (IAT)

**Figure 1:**
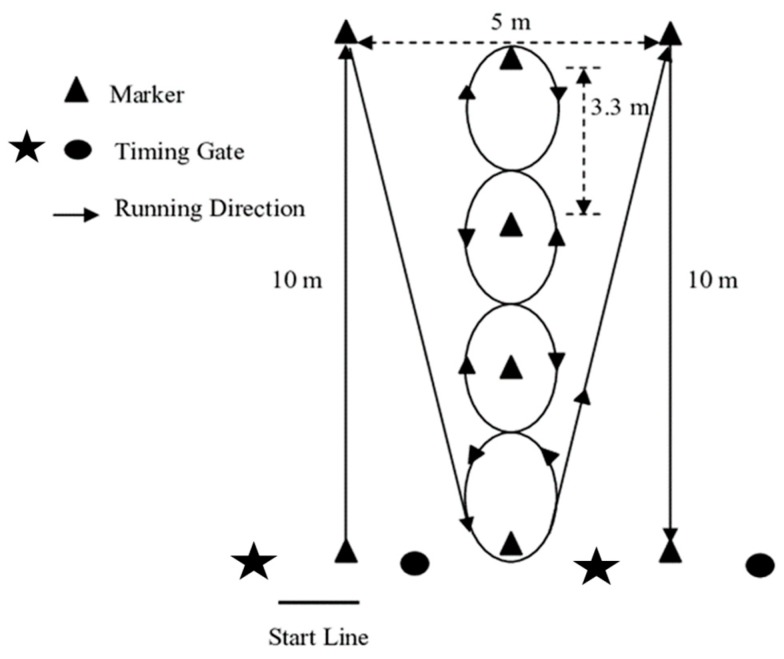
The Illinois agility test (IAT).

The dimensions and route direction for the IAT are shown in Figure 1 and was conducted according to previous research; the methods have been described in these studies [5,34,35]. Two pairs of single-beam timing gates were used at the start and finish lines. The height of the gates was set at 0.93 m [59,61], and they were positioned 1.93 m apart. A standing start 50 cm behind the start line was used. Subjects were instructed not to step over the markers but run around them, and were to follow the prescribed route throughout the trial.

### 2.7. 505 Test

**Figure 2:**
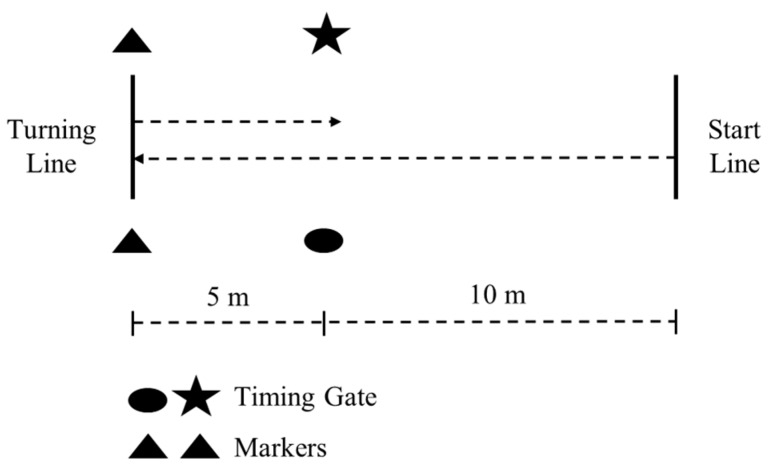
The 505 test.

The methodology for the 505, as shown in Figure 2, was conducted per established methods, which have been described in detail [5,31,52,53]. A pair of timing gates set at a height of 0.93 m [59,61], and width of 2.84 m was used. The turning line was indicated by a line marked on the floor and with cones. Two trials were recorded for turns off the left and right foot, the order of which was randomized amongst the subjects. COD deficit for each leg was calculated via the formula: 505 time–10 m time [29]. The 10 m time was taken from the 0–10 m split from the linear sprint test, which follows standard procedures [7,10,29,30,31,32,54].

### 2.8. Vertical Jump (VJ)

The countermovement vertical jump measured lower-body power in the vertical plane. The VJ was measured via a Jump Station apparatus (EPIC Athletic Performance, Lincoln, NE, USA), and was conducted according to established methods described elsewhere [20,22,52,53,62]. Peak anaerobic power measured in watts (PAPw) from the VJ was calculated for the best trial by the equation: PAPw = (60.7⋅VJ height (cm)) + (45.3·body mass (kg)) − 2055 [63]. PAPw was calculated relative to body mass to provide a power-to-body mass ratio (P:BM) via the equation: P:BM = PAPw·BM^−1^ [7,12,62,64].

### 2.9. Isometric Midthigh Pull (IMTP)

To perform the IMTP, subjects stood on a force plate (AMTI, Watertown, MA, USA) within a power rack (Rogue Fitness, Columbus, OH, USA). The power rack allowed the bar to be fixed at the mid-point of the thigh (halfway between the iliac crest and the midpoint of the patella) for each subject. Subjects were instructed to grip the bar in a position similar to that of a second pull of a power clean, with an upright trunk position and so that their shoulders were in line with the bar, in their preferred position for the pull [65]. Subjects performed two, 5 s trials of the IMTP. In the advent of a difference in peak force of in excess of 250 N between the two trials, a third trial was performed [66]. A countdown of “3, 2, 1, take the weight” was given to the subjects before they initiated the pull. Subjects were instructed to pull as hard as possible, while driving their feet as hard as possible into the force plate. Data from the force plate was sampled at 1000 Hz and converted from analog to digital. This followed procedures established in the literature that led to the production of reliable IMTP data [67]. Digital signals were processed using a custom program through LabVIEW 2015 software (Version 15.0.1; National Instruments, Austin, TX, USA) where peak force was recorded and used for analysis. Relative force was also calculated according to the equation: relative force (N·kg^−1^) = peak force∙body weight in kg^−1^.

### 2.10. Statistical Analysis

All statistical analyses were computed using the Statistics Package for Social Sciences (Version 24.0; IBM Corporation, New York, NY, USA). Descriptive statistics (mean ± standard deviation [SD]) were calculated for each test parameter, and chi-square goodness of fit tests were conducted to check data distribution. Independent samples t-tests were utilized to determine whether differences in the performance tests existed between males and females and to confirm the use of sex as a control variable. An alpha level of *p* < 0.05 was required for significance. The relationships between the COD speed tests (505, COD deficit, and IAT) with the 20 m sprint intervals, jumps tests, and IMTP was investigated via partial correlations controlling for sex (*p* < 0.05). The correlation strength was designated as: an *r* between 0 to 0.3, or 0 to −0.3, was considered small; 0.31 to 0.49, or −0.31 to −0.49, moderate; 0.5 to 0.69, or −0.5 to −0.69, large; 0.7 to 0.89, or −0.7 to −0.89, very large; and 0.9 to 1, or −0.9 to −1, near perfect for relationship prediction [68]. Stepwise linear regression analyses (*p* < 0.05), with sex as a control variable, were conducted for the COD speed tests (each acted as a dependent variable) to illustrate whether a linear sprint interval, jump test variable, or strength variable predicted performance. This approach was undertaken due to the exploratory nature of this study and is similar to previous research [23].

## 3. Results

The chi-square goodness of fit analysis for all the variables indicated an expected data distribution when weighted by sex (χ^2^ (25–42) = 7.11–34.51, *p* = 0.15–1.00). Descriptive data for the combined, males, and females is shown in Table 1. There were significant differences between the sexes in all tests except for the left-leg COD deficit, which confirmed the need to control for sex in the correlation and regression analyses. 

The partial correlation data is shown in Table 2. For the 505 from each leg and IAT, there were significant relationships with all tests except PAPw and absolute strength measured by the IMTP. Relationships with the linear sprint intervals were positive, and negative with the jump tests and relative IMTP strength; relationship strength ranged from moderate-to-large. For the COD deficit for each leg, there were only significant negative relationships with the LJ, which were moderate in strength. 

The stepwise linear regression data is shown in Table 3. Sex was not entered into the model for the left-leg COD deficit, and thus this predictive equation was not generated. All predictive relationships were significant at *p* < 0.01, and sex was involved in all significant relationships. The 0–20 m sprint interval and left-leg LJ predicted the left- and right-leg 505, with an explained variance of 70% and 77%, respectively. The right-leg COD deficit was predicted by the left-leg LJ, with an explained variance of 27%. The IAT was predicted by the 0–20 m sprint interval, right-leg LJ, and relative strength measured by the IMTP, with an explained variance of 84%.

## 4. Discussion

The current study investigated how linear speed, lower-body power, and lower-body isometric strength may influence COD speed as measured by shorter and longer distance tests in college-aged individuals. This information is important, as collegiate club-sport [24,25] and tactical recruits [26,27,28] may not have the training time of other athletes. Understanding the physical qualities that could most influence COD speed improvements over shorter and longer distances could enhance training efficiency. The results indicated that for both the shorter (505) and longer (IAT) COD tests, faster linear speed, and greater lower-body power and relative isometric strength, related to faster COD performance. As hypothesized, the relationships tended to be stronger for the IAT. However, there were limited relationships between the COD deficit and the performance tests. The results from this study have implications for the training of college-aged individuals, especially those that need to train efficiently when developing their COD speed.

The 505 was used in this study as a measure of shorter COD speed. Previous 505 research has documented relationships between linear speed over different distances [6,7,29], lower-body power as measured by multidirectional jump tests [6,7,8], and lower-body strength [6,9]. The results from this study supported these findings. All sprint intervals and the jump tests correlated with the 505 from each leg, while the 0–20 m sprint interval and left-leg LJ predicted the 505. These data could have been influenced by the degree of linear sprinting (10 m about the 180° turn) still present within the 505 [4,29]. Nonetheless, these data illustrate the potential importance of linear sprinting ability and lower-body strength and power to shorter COD performance in recreationally-active individuals. Improvements in these qualities could influence athletic and occupational tasks that require shorter COD actions. Interestingly, P:BM but not PAPw, and relative IMTP but not absolute IMTP, correlated with the 505. These results indicate that males and females typical of collegiate club-sport [8,10,11,12] and tactical recruit [13,14,15,16,17,18] athletes should not just improve their lower-body strength and power to enhance their shorter COD speed, but ensure they can express high force and power relative to their body mass.

As 505 performance can be influenced by linear speed, the COD deficit may provide an alternate measure of shorter COD performance [4,29]. Previous research has found limited relationships between physical fitness assessments and the COD deficit [8,29,30,31]. The current results provided further support to these studies. Only the LJ demonstrated significant relationships to the COD deficit, with the left-leg LJ predicting the right-leg COD deficit (although the predictive relationship was low; 27% explained variance when combined with sex). These relationships could relate to the actions inherent within the 505 (i.e., the lateral 180° direction change). Nevertheless, given the limited relationships with the performance tests, this may suggest that technical qualities (i.e., COD biomechanics) may influence the COD deficit more than an individual’s physical qualities [69]. Considering this, and within the context of this study where individuals may be more time-restricted with their training [24,25,26,27,28], shorter COD speed could be initially targeted via improvements in linear speed and lower-body power and strength. Once this foundation is present and should the opportunity arise, more directed technique training could be adopted.

The IAT has been used to assess COD speed in athletic [33,34,35,36,37,38,39,40], law enforcement/public safety [41,42,43], and military [44,45,46] populations. COD speed tests that have a duration above 6 s (e.g., Arrowhead test, T-test) have been found to have significant relationships with linear speed tests [12,47,48]. This is most likely due to the greater demands on linear sprinting. Further to this, given that sprint performance often exhibits strong relationships with jumping ability as both utilize the stretch-shortening cycle [70], longer distance COD speed tests often demonstrate relationships with multidirectional jump performance [12,47]. This was reflected in the results from this study. All linear sprint intervals and jump tests correlated with the IAT, and the IAT was predicted by the 0–20 m sprint interval, right-leg LJ, and relative IMTP. Similar to the 505 results, P:BM and relative IMTP correlated with the IAT, while PAPw and absolute IMTP did not. These data indicate the potential value of enhancing linear speed, and lower-body power and strength relative to body mass, for males and females that need to perform longer distance COD speed efforts. This has particular relevance in the occupational field, as tasks such as simulated foot pursuits can be performed over distances of approximately 70–90 m, with multiple direction changes [19,20,21,22,23]. To efficiently improve longer distance COD speed efforts, linear speed, lower-body strength, and lower-body power training should be used by males and females. Although this may seem a straightforward recommendation, these training approaches are not often adopted in the occupational field [22,23], even with a greater focus nowadays on the tactical athlete.

There are study limitations that should be acknowledged. Only two COD speed tests were analyzed in this study; it would be of interest to investigate other shorter (e.g., pro-agility shuttle, three-cone drill) or longer (e.g., Arrowhead, T-test) assessments used to measure COD ability [5]. Further to this, the relationships between physical qualities and direction change performance through different turn angles (e.g., 45° vs. 90° vs. 180°) should be considered. Nimphius et al. [4] has noted that the angle of a direction change will influence COD speed, and this is likely linked to the physical qualities required for faster performance. Only bilateral isometric strength was measured in this study. As unilateral and dynamic strength should have some influence on COD speed, greater analysis of the impact of lower-body strength on shorter and longer COD performance could be conducted. Lastly, the sample analyzed were recreationally-trained, college-aged males and females, and not a specific athletic population. Despite this, the sample has great utility, and as noted earlier, is similar to collegiate club-sport [8,10,11,12] and tactical recruit [13,14,15,16,17,18] athletes. Accordingly, the results may be inferred across lower-level college athletes from different sports and individuals from different occupations (e.g., first responders, military), as these groups all are required to perform shorter and longer distance COD tasks.

## 5. Conclusions

The results from this study indicated that in recreationally-trained, college-aged individuals, linear speed over 20 m, lower-body power measured via multidirectional jumping, and lower-body isometric strength related to shorter (505) and longer (IAT) COD performance. Moreover, P:BM and relative IMTP was more important than absolute power and force generation for these COD tasks. For those individuals who may have limited training time, such as collegiate club-sport [24,25] and tactical recruit [26,27,28] athletes, specific targeting of linear sprinting, and relative lower-body power and strength could lead to enhanced shorter and longer COD performance. Finally, the COD deficit, which may provide an alternate measure of shorter COD ability, did not relate to most of the speed, power, and strength tests. Improvements in this quality may require more targeted technique development.

## Figures and Tables

**Table 1:** Descriptive data (mean ± SD) for combined, males, and females in the 505 and change-of-direction (COD) deficit for the left (L) and right (R) legs, IAT, 0–5 m, 0–10 m, and 0–20 m sprint intervals, vertical jump (VJ), peak anaerobic power measured in watts (PAPw), power-to-body mass ratio (P:BM), standing broad jump (SBJ), lateral jump (LJ) for the left (L) and right (R) legs, and absolute and relative isometric midthigh pull (IMTP).

Variables	Combined (n = 43)	Males (n = 21)	Females (n = 22)	*p*
505 L (s)	2.71 ± 0.29	2.54 ± 0.19	2.90 ± 0.25 *	<0.01
COD Deficit L (s)	0.70 ± 0.19	0.67 ± 0.19	0.74 ± 0.18	0.18
505 R (s)	2.73 ± 0.30	2.53 ± 0.16	2.94 ± 0.26 *	<0.01
COD Deficit R (s)	0.72 ± 0.18	0.66 ± 0.16	0.78 ± 0.17 *	0.02
IAT (s)	17.87 ± 1.68	16.78 ± 0.99	19.02 ± 1.49 *	<0.01
0–5 m (s)	1.18 ± 0.12	1.10 ± 0.07	1.26 ± 0.12 *	<0.01
0–10 m (s)	2.01 ± 0.21	1.87 ± 0.11	2.16 ± 0.19 *	<0.01
0–20 m (s)	3.54 ± 0.40	3.26 ± 0.20	3.83 ± 0.34 *	<0.01
VJ (cm)	49.53 ± 13.17	59.05 ± 10.54	39.57 ± 6.62 *	<0.01
PAPw (w)	4318.31 ± 1108.12	5203.36 ± 637.03	3391.12 ± 623.10 *	<0.01
P:BM (w·kg^−1^)	57.83 ± 10.08	64.59 ± 8.19	50.75 ± 6.31 *	<0.01
SBJ (m)	1.92 ± 0.41	2.23 ± 0.25	1.59 ± 0.25 *	<0.01
LJ L (m)	1.44 ± 0.30	1.62 ± 0.25	1.26 ± 0.22 *	<0.01
LJ R (m)	1.43 ± 0.32	1.64 ± 0.25	1.21 ± 0.21 *	<0.01
IMTP (N)	1936.29 ± 488.39	2324.10 ± 275.05	1530.01 ± 287.58 *	<0.01
Relative IMTP (N·kg^−1^)	25.91 ± 4.20	28.74 ± 2.61	22.95 ± 3.44 *	<0.01

* Significantly (*p* < 0.05) different from the males.

**Table 2:** Partial correlations controlling for sex in recreationally-trained men and women between the 505 and COD deficit for the left (L) and right (R) legs and IAT, with the 0–5 m, 0–10 m, and 0–20 m sprint intervals, VJ, PAPw, P:BM, SBJ, LJ for the left (L) and right (R) legs, and absolute and relative IMTP (n = 43). *r*: correlation coefficient; *p*: significance.

Variables		505 L	COD Deficit L	505 R	COD Deficit R	IAT
0–5 m	*r* *p*	0.46 * <0.01	−0.25 0.11	0.58 * <0.01	−0.14 0.40	0.57 * <0.01
0–10 m	*r* *p*	0.57 * <0.01	−0.15 0.35	0.64 * <0.01	−0.08 0.61	0.64 * <0.01
0–20 m	*r* *p*	0.64 * <0.01	−0.03 0.86	0.71 * <0.01	0.04 0.82	0.74 * <0.01
VJ	*r* *p*	−0.43 * 0.01	−0.18 0.26	−0.46 * <0.01	−0.22 0.16	−0.45 * <0.01
PAPw	*r* *p*	−0.17 0.28	−0.15 0.35	−0.26 0.10	−0.27 0.08	−0.24 0.13
P:BM	*r* *p*	−0.51 * <0.01	−0.24 0.14	−0.51 * <0.01	−0.24 0.13	−0.51 * <0.01
SBJ	*r* *p*	−0.60 * <0.01	−0.18 0.26	−0.61 * <0.01	−0.20 0.20	−0.66 * <0.01
LJ L	*r* *p*	−0.62 * <0.01	−0.43 * <0.01	−0.60 * <0.01	−0.44 * <0.01	−0.67 * <0.01
LJ R	*r* *p*	−0.55 * <0.01	−0.34 * 0.03	−0.57 * <0.01	−0.39 * 0.01	−0.66 * <0.01
IMTP	*r* *p*	−0.15 0.36	−0.15 0.34	−0.25 0.11	−0.30 0.06	−0.30 0.06
Relative IMTP	*r* *p*	−0.50 * <0.01	−0.18 0.25	−0.54 * <0.01	−0.24 0.13	−0.63 * <0.01

* Significant (*p* < 0.05) relationship between the two variables.

**Table 3:** Stepwise linear regression analysis between the COD speed tests (505 and COD deficit for the left [L] and right [R] legs, IAT) and VJ, PAPw, P:BM, SBJ, left- and right-leg LJ, and absolute and relative IMTP in recreationally-trained men and women (n = 43).

Variables		*R*	*R* ^2^	Adjusted *R*^2^
505 L	Sex	0.64 **	0.41	0.39
	Sex, 0–20 m sprint	0.81 ***	0.65	0.64
	Sex, 0–20 m sprint, Left-leg LJ	0.85 ***	0.72	0.70
505 R	Sex	0.69 **	0.48	0.47
	Sex, 0–20 m sprint	0.86***	0.73	0.73
	Sex, 0–20 m sprint, Left-leg LJ	0.89 ***	0.77	0.77
COD Deficit R	Sex	0.36 *	0.13	0.11
	Sex, Left-leg LJ	0.55 **	0.30	0.27
IAT	Sex	0.68 **	0.45	0.44
	Sex, 0–20 m sprint	0.87 ***	0.76	0.74
	Sex, 0–20 m sprint, Right-leg LJ	0.91 ****	0.82	0.81
	Sex, 0–20 m sprint, Right-leg LJ, Relative IMTP	0.93 ****	0.86	0.84

Note: no variables were entered into the predictive model for the left-leg COD deficit. * = moderate, ** = large, *** = very large, **** = near perfect.

## Abbreviations

COD	Change-of-direction
m	Meter
VJ	Vertical jump
LJ	Lateral jump
*r*	Correlation coefficient
SBJ	Standing broad jump
IMTP	Isometric midthigh pull
s	Seconds
IAT	Illinois agility test
kg	Kilograms
cm	Centimeters
PAPw	Peak anaerobic power measured in watts
P:BM	Power-to-body mass ratio
N	Newtons
SD	Standard deviation
*p*	Significance
w	Watts
w·kg^−1^	Watts per kilogram body mass
N·kg^−1^	Newtons per kilogram body mass
L	Left
R	Right
